# Enhanced immune responses following heterologous vaccination with self-amplifying RNA and mRNA COVID-19 vaccines

**DOI:** 10.1371/journal.ppat.1010885

**Published:** 2022-10-04

**Authors:** Tamara Elliott, Hannah M. Cheeseman, Abbey B. Evans, Suzanne Day, Leon R. McFarlane, Jessica O’Hara, Mohini Kalyan, Fahimah Amini, Tom Cole, Alan Winston, Sarah Fidler, Katrina M. Pollock, James A. Harker, Robin J. Shattock

**Affiliations:** 1 Department of Infectious Disease, Imperial College London, United Kingdom; 2 Imperial College Healthcare NHS Trust, London, United Kingdom; 3 Imperial College NIHR BRC, London, United Kingdom; 4 National Heart and Lung Institute, London, United Kingdom; Chang Gung University, TAIWAN

## Abstract

The optimal vaccination strategy to boost responses in the context of pre-existing immune memory to the SARS-CoV-2 spike (S) glycoprotein is an important question for global public health. To address this, we explored the SARS-CoV-2-specific humoral and cellular immune responses to a novel self-amplifying RNA (saRNA) vaccine followed by a UK authorised mRNA vaccine (BNT162b2) in individuals with and without previous COVID-19, and compared these responses with those who received an authorised vaccine alone. 35 subjects receiving saRNA (saRNA group) as part of the COVAC1 clinical trial and an additional 40 participants receiving an authorised SARS-CoV-2 vaccine only (non-saRNA group) were recruited. Antibody responses were measured by ELISA and a pseudoneutralisation assay for wildtype, Delta and Omicron variants. Cellular responses were measured by IFN-ƴ ELISpot and an activation induced marker (AIM) assay. Approximately 50% in each group had previous COVID-19 prior to vaccination, confirmed by PCR or antibody positivity on ELISA. All of those who received saRNA subsequently received a full course of an authorised vaccine. The majority (83%) of those receiving saRNA who were COVID-19 naïve at baseline seroconverted following the second dose, and those with previous COVID-19 had an increase in antibody titres two weeks following saRNA vaccination (median 27-fold), however titres were lower when compared to mRNA vaccination. Two weeks following the 2^nd^ authorised mRNA vaccine dose, binding and neutralising antibody titres were significantly higher in the saRNA participants with previous COVID-19, compared to non-saRNA, or COVID-19 naive saRNA participants. Cellular responses were again highest in this group, with a higher proportion of spike specific CD8+ than CD4+ T cells when compared to those receiving the mRNA vaccine only. These findings suggest an immunological benefit of increased antigen exposure, both from natural infection and vaccination, particularly evident in those receiving heterologous vaccination with saRNA and mRNA.

## Author summary

With the continuing emergence of SARS-CoV-2 variants of interest and of concern, there is an ever-growing population of people recovered from or immunised against COVID-19. The optimal vaccination strategies are still not established, but mounting evidence suggests enhanced protection from booster vaccination, and vaccination following natural infection (hybrid immunity). In this study we have compared immune responses in individuals who have received a novel self-amplifying RNA (saRNA) COVID-19 vaccine who have then subsequently received a UK authorised vaccine, and a group who received an authorised vaccine alone. Half the participants in each group had recovered from COVID-19. We found a superior immune response in those who had previous COVID-19 and received saRNA plus a UK authorised vaccine with regards to binding and neutralising antibody production, breadth of neutralisation against variants of concern (Delta and Omicron lineage variants) and T-cell responses. These findings suggest an immunological benefit of combining previous natural infection with vaccination against COVID-19 using a combination of different vaccine platforms.

## Introduction

The effectiveness of COVID-19 vaccine-induced immunity in population level control of the pandemic is limited by focusing the immune response on a single viral protein, the SARS-CoV-2 spike (S) and by the need to continuously re-immunise in mass vaccination programmes. Having completed booster immunisation, some countries have introduced a fourth dose with an aim to limit severe disease and hospitalisation caused by the highly transmissible Omicron variants. The high level of transmissibility of current circulating SARS-CoV-2 variants means that over time there is an ever-increasing proportion of individuals who have been exposed to the virus in addition to vaccine induced immunity. This hybrid immunity, may be adjunctive to vaccine exposure [1] and could potentially be leveraged in vaccination programmes to more effectively control the pandemic. Given the need for on-going and widespread COVID-19 vaccination programmes, vaccines that can be relatively cheaply produced at scale and induce optimal immunity in this landscape would be welcome, and RNA vaccines may fit this brief.

Amongst the first COVID-19 vaccines to receive emergency use authorisation are messenger RNA (mRNA) vaccines, which demonstrated ~95% efficacy against symptomatic COVID-19 in clinical trials [2,3]. Although mRNA vaccine technology is not new, it is in its infancy, with BNT162b2 (Pfizer/BioNTech) being the first mRNA vaccine to be licensed for use against any infectious pathogen [4]. As with conventional mRNA vaccines, self-amplifying RNA (saRNA) vaccines contain the genetic material for a viral protein, but also encode a viral replicase (usually an alphavirus) with the gene of interest replacing the structural genes of the virus [5,6]. The replicase amplifies the complete saRNA sequence followed by preferential amplification of the gene of interest, which is driven by a sub-genomic promoter [5]. Due to this self-amplification, the intended result is many more copies of antigen over a longer period compared with mRNA vaccines, and so, theoretically, saRNA vaccines may be given at lower doses to get the same or enhanced immune response [5,6]. The use of these vaccines can potentially reduce vaccine cost and therefore increase coverage. No saRNA vaccines have been licenced for use to date but they have been shown to be immunogenic in animal models for many infectious diseases, including influenza A and B viruses [7], respiratory syncytial virus (RSV) [8], and, more recently, SARS-CoV-2 [9–14].

The safety and immunogenicity of one such saRNA vaccine is being trialled in the COVAC1 Phase I/II clinical trial (ISRCTN17072692). This vaccine uses the non-infectious Venezuelan equine encephalitis virus (VEEV) replicon backbone encoding non-structural proteins required for self-amplification, with the prefusion stabilised SARS-CoV-2 spike protein in place of the structural genes of the alphavirus. This presentation of spike and the lipid nanoparticle used to encapsulate the saRNA is identical to that used in BNT162b2 [15]. Results from pre-clinical studies in mice demonstrated high titre binding antibodies post-immunisation with vaccine doses ranging from 0.01–10 μg [9]. The mice had higher concentrations of IgG than human COVID-19 convalescent samples, with IgG titres correlating with viral neutralisation. Stimulated splenocytes from vaccinated mice also demonstrated high IFN-γ secretion as quantified by ELISpot [9].

In the COVAC1 Phase I dose ranging trial, the safety and immunogenicity of six dose levels was assessed (from 0.1 μg to 10.0 μg) based on pre-clinical data. Initial dose ranging was at 3 doses (0.1 μg, 0.3 μg and 1 μg), but due to disappointing immunogenicity at these low doses [16], a second dose escalation took place with a further 3 doses (2.5 μg, 5 μg, and 10 μg). Data from the dose escalation and evaluation trial in 192 individuals demonstrated vaccine safety and reactogenicity comparable to other available COVID-19 vaccines, and seroconversion rates as measured by ELISA were highest at the 10 μg dose tested [16]. Following the dose escalation and evaluation portion of the COVAC1 trial, subjects were recruited for expanded safety evaluation across multiple sites. These participants, who were healthy adults aged 18–75 years, received an initial vaccine dose of 1 μg (the highest dose tested in the initial dose escalation), but were offered to delay their second 1 μg dose (due at 28 days) to receive a higher 10 μg dose (the highest dose tested in the second dose escalation). These participants subsequently received an authorised COVID-19 vaccine. A previous history of COVID-19 was an exclusion criterion except at one recruiting site (Imperial College Healthcare NHS Trust (ICHT)), where participants were specifically selected for having had PCR- or antibody-confirmed previous COVID-19 infection.

In this study we have explored the immune responses in COVAC1 participants receiving two doses of saRNA vaccine and subsequently an authorised COVID-19 mRNA vaccine (BNT162b2) and compared this to those receiving mRNA vaccine alone. With the emergence of variants of concern (VoC) we have also explored the breadth of neutralising capacity in these participants. We hypothesised that the unique kinetic properties of the saRNA vaccine could be used to deliver improved boosting and hybrid immunity in the setting of the current COVID-19 vaccination programme.

## Results

### Recruitment and demographics

**Fig 1:**
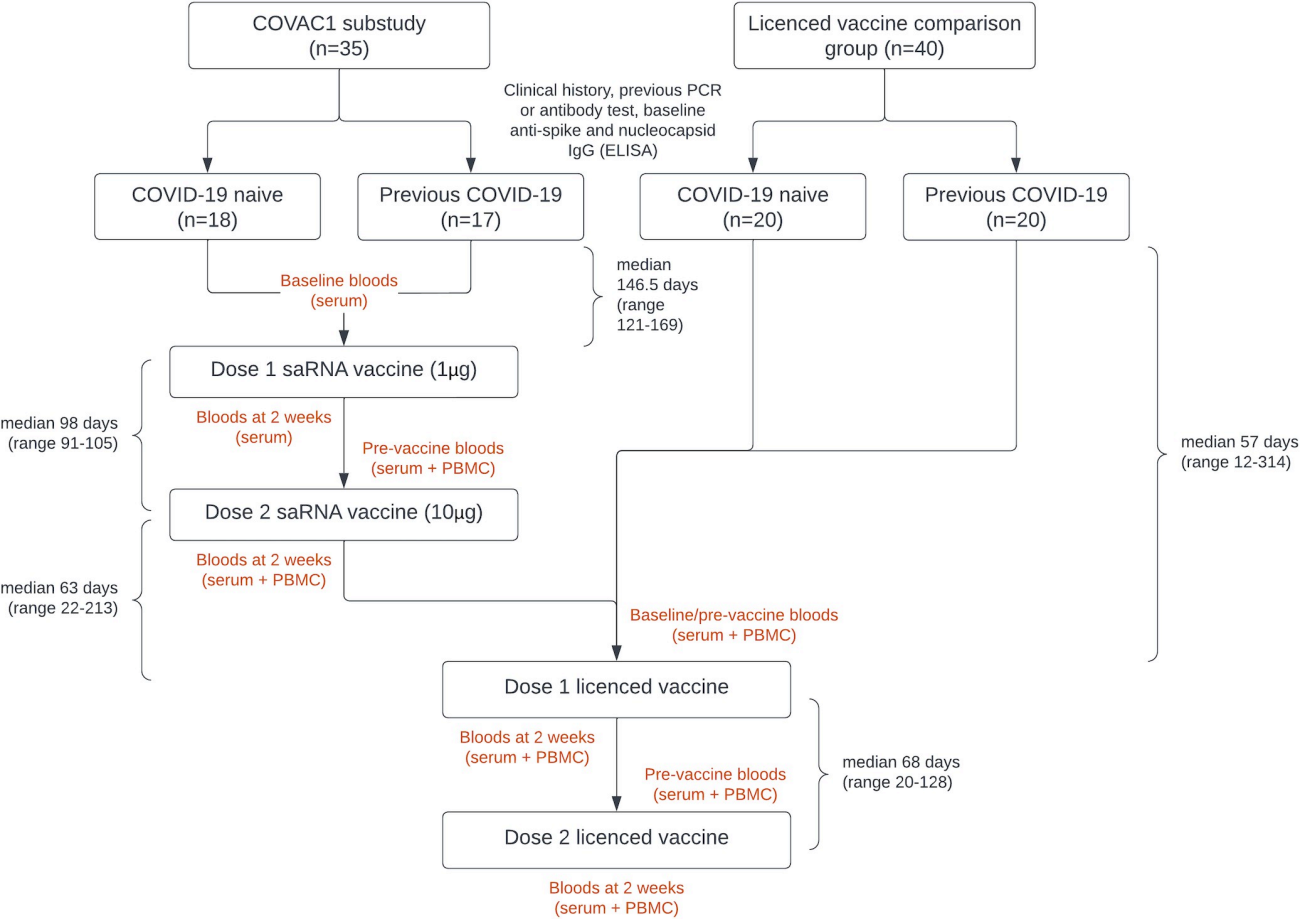
Simplified study schematic.

Of the 37 COVAC1 participants in the expanded safety evaluation at ICHT, 35 consented to take part in a sub-study (saRNA group) in which additional samples for cellular immunological responses were collected (Fig 1). Of these, 15 were known to have previous COVID-19 based on a previous positive PCR or a diagnostic antibody test performed as part of clinical care. 40 participants were recruited into a comparison group who received authorised mRNA vaccine only (non-saRNA group), of which 18 were known to have previous COVID-19. In conjunction with baseline ELISA data, 17/35 in the saRNA (COVAC1) group and 20/40 in the non-saRNA comparison group were defined as having previous infection at the time of enrolment (Table 1).

**Table 1:** Participant demographics, infection and vaccine history in the saRNA (COVAC1) and non-saRNA participants by infection status.

	saRNA followed by licensed vaccine (saRNA group) (n = 35)		Licensed vaccine only (non-saRNA group) (n = 40)		
	Previous COVID-19 (n = 17)	COVID-19 naïve (n = 18)	Previous COVID-19 (n = 20)	COVID-19 naïve (n = 20)	p value
Vaccine received BNT162b2	17 (100%)	8 (44%)	19 (95%)	20 (100%)	<0.0001
ChAdOx nCoV-19	0	10 (56%)	1 (5%)	0	
Age (median, range) BNT162b2 recipients*	30 (21–58) *30 (21–58)*	52 (28–73) *47*.*5 (28–67)*	32 (24–48) *31 (24–48)*	32.5 (25–70) *32*.*5 (25–70)*	0.0002 *0*.*1547*
Male sex BNT162b2 recipients*	5 (29%) *5 (29%)*	10 (56%) *4 (50%)*	7 (35%) *7 (37%)*	5 (25%) *5 (25%)*	0.2300 *0*.*6093*
Healthcare worker BNT162b2 recipients*	14 (82%) *14 (82%)*	7 (39%) *5 (63%)*	17 (85%) *17 (89%)*	12 (60%) *12 (60%)*	0.0051 *0*.*1442*
Known previous COVID-19 Asymptomatic Mild Moderate Severe	15 (88%) 4 (27%) 6 (40%) 4 (27%) 0	n/a	18 (90%) 2 (11%) 7 (39%) 9 (50%) 0	n/a	0.1810
Days since COVID-19 diagnosis to 1^st^ vaccine dose (median, range)	146.5 (121–169)	n/a	57 (12–314)	n/a	0.0343
COVID-19 during 1^st^ wave (March-May 2020, Wuhan-hu-1) if known	15 (100%)	n/a	6 (33%)	n/a	0.0001
COVID-19 during 2^nd^ wave (Sept 2020-Feb 2021, predominantly Alpha variant) if known	0	n/a	12 (67%)	n/a	
Days since COVID-19 diagnosis to 1^st^ authorised vaccine dose (1^st^ wave only)	284.5 (249–319)	n/a	303 (279–314)	n/a	0.2520
Days since COVID-19 diagnosis to 1^st^ authorised vaccine dose (2^nd^ wave only)	n/a	n/a	41 (12–109)	n/a	n/a

*Demographics for participants receiving BNT162b2 presented separately as those receiving ChAdOx nCoV-19 excluded from final analysis

All saRNA participants subsequently received a UK authorised vaccine, with 25 receiving BNT162b2, and 10 receiving the ChAdOx nCoV-19 adenoviral vector vaccine. In the non-saRNA group, all but one participant received BNT162b2 (Table 1). Only those receiving the BNT162b2 mRNA vaccine were included in the analysis.

### Binding and neutralising antibody

**Fig 2:**
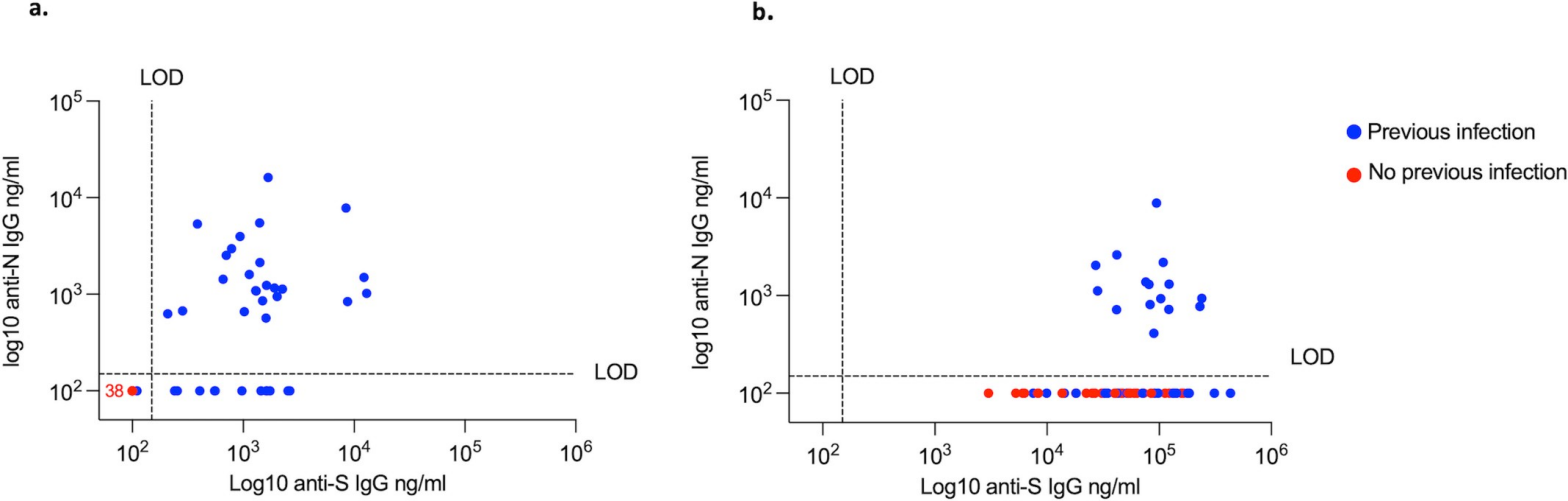
Anti-spike versus anti-nucleocapsid IgG. **2a**. Baseline (pre-vaccination) anti-S and anti-N IgG in COVID-19 experienced (blue, n = 37) and COVID naïve (red, n = 38) participants as measured by ELISA. **2b**. Anti-S and anti-N IgG two weeks following the 2^nd^ UK authorised vaccine dose in COVID-19 experienced (n = 36) and naïve (n = 33). LOD; level of detection.

At baseline, 17/35 (49%) and 12/35 (34%) in the saRNA group and 20/40 (50%) and 13/40 (33%) in the non-saRNA group had detectable antibodies against Spike (S) and Nucleocapsid (N), respectively. One participant in the non-saRNA group with a previous positive PCR did not have detectable antibodies against S or N at baseline (Fig 2A). At the final time-point reported here (2 weeks following the second dose authorised vaccine) none of the participants defined as COVID-19 naïve developed antibodies against N (Fig 2B).

**Fig 3:**
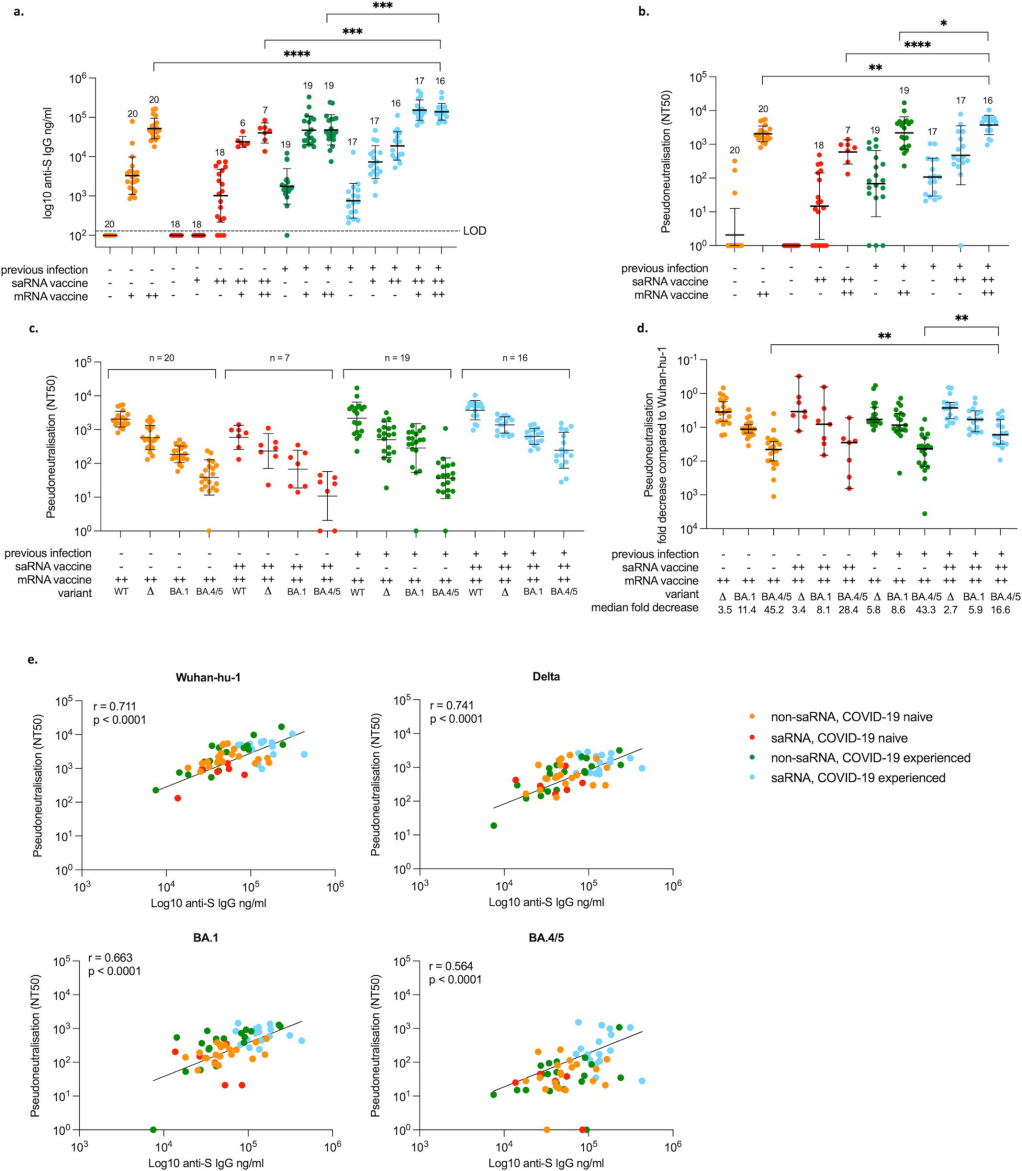
Binding and neutralising antibody responses. **3a**. Antibodies against SARS-CoV-2 Wuhan-hu-1 spike protein as measured by ELISA at baseline and two weeks following dose 1 and 2 of saRNA and mRNA vaccines in saRNA (COVAC1) participants (red and blue) and at baseline and two weeks following dose 1 and 2 of mRNA vaccines in non-saRNA participants (orange and green). **3b**. Neutralising antibodies against SARS-CoV-2 Wuhan-hu-1 measured using pseudovirus at baseline and two weeks following the 2^nd^ dose of saRNA and mRNA vaccines in saRNA participants (red and blue) and two weeks following the 2^nd^ dose of mRNA vaccine in non-saRNA participants (orange and green). **3c**. Neutralising antibodies against SARS-CoV-2 Wuhan-hu-1, Delta and Omicron BA.1 and BA.4/5 using pseudovirus two weeks following the second mRNA vaccine dose and the differences between groups. **3d**. Fold decrease in neutralisation against Delta and Omicron BA.1 and BA.4/5 compared to Wuhan-hu-1 (y axis inverted) **3e**. Correlation between binding antibodies against SARS-CoV-2 Wuhan-hu-1 spike protein (ELISA) and neutralisation (pseudovirus) against Wuhan-hu-1, Delta and Omicron BA.1 and BA.4/5 two weeks following the second mRNA vaccine dose. Number of samples included in the analysis indicated on graphs. Geometric mean titres (GMT) and standard deviation (sd) are shown. Differences between groups determined by Kruskall-Wallis and tested using Mann-Whitney. Median fold decrease within groups by Wilcoxon matched pairs signed rank test. Correlations by Spearman’s rank correlation coefficient. -, no exposure; +, single exposure; ++, two exposures; LOD, level of detection; WT, wildtype; Δ, Delta. Significant values displayed: **** p<0.0001; *** p<0.001; ** p<0.01; * p<0.05; ns, non-significant.

Participants in the saRNA group (COVAC1) received an initial 1 μg dose of saRNA vaccine. All these participants elected to delay their second dose while the additional dose escalation (2.5 μg, 5 μg and 10 μg) took place, and therefore all subsequently received a higher 10 μg second dose with a median interval of 98 days (range 91–105) between doses (Fig 1). Following the first dose, 1/18 participants defined as COVID-19 naïve had seroconverted by anti-S IgG ELISA by the time of their second dose. This increased to 15/18 (83%) two weeks following their 2^nd^ 10 μg dose (geometric mean titre (GMT) 1,623 ng/mL (sd±3.43) for those that did seroconvert) (Fig 3A). The saRNA participants with previous COVID-19 all had detectable antibodies against S at baseline (GMT 758 ng/mL, sd±2.77), and there was an increase in titres two weeks following their first saRNA dose (GMT 7,311 ng/mL (sd±2.61), median 6.7-fold increase), with a further increase seen after the 2^nd^ dose (GMT 18,862 ng/mL (sd±2.31), median 27-fold increase from baseline) (Fig 3A).

All participants in the saRNA group became eligible for an authorised vaccine during the study. The interval between 2^nd^ saRNA vaccine dose and the 1^st^ authorised vaccine dose was a median of 63 days (range 22–213), and varied considerably, with healthcare workers and older participants becoming eligible for vaccination first according to UK guidelines at the time. By chance none of the 10 participants who received ChAdOx nCoV-19 in the saRNA group were defined as having previous infection. The timepoints following ChAdOx nCoV-19 have been excluded from analysis since these participants all fall within the same COVID-19 naïve group, and it has been shown that the antibody concentrations following this adenoviral vector vaccine are less than those observed following mRNA [17]. Of the 35 COVAC1 participants in the saRNA group, we were able to collect follow up samples for 28 subjects two weeks after their 1^st^ and 2^nd^ authorised vaccine doses.

After the first dose of BNT162b2, all COVID-19 naïve participants in the saRNA group had an increase in antibody titres (GMT 23,865 ng/mL (sd±1.38)), including those who did not seroconvert following saRNA vaccination. There was a further increase in titres 2 weeks following their second authorised vaccine dose (data for n = 7, GMT 39,259 ng/mL, sd±1.90) (Fig 3A). Participants with previous infection in the saRNA group who subsequently received BNT162b2 (n = 17) saw a robust increase in antibody titres following a single dose (GMT 152,443 ng/mL (sd±1.82), median-6.8 fold) with no further increase following the second dose (Fig 3A).

For those in the non-saRNA group, all participants without previous infection seroconverted after their first authorised vaccine dose (GMT 3,283 ng/mL (sd±2.98)) with further increase following the second dose (GMT 52,010 ng/mL (sd±1.83), median 17.7-fold increase) (Fig 3A). Prior to vaccination, the GMT for those with previous infection was 1,381 ng/mL (sd±6.79), with one participant seronegative. Following a single dose of BNT162b2 the GMT increased to 46,991 ng/mL (sd±2.29) (median 23.4-fold increase) with again no significant increase following the second dose (Fig 3A).

Of the COVAC1 participants in the saRNA group who were COVID-19 naïve, neutralising antibodies against Wuhan-hu-1 were detected in 12/18 (67%) two weeks following the second saRNA vaccine dose (geometric mean (GM) 50% neutralisation titre (NT50) 57, sd±4.1). Following the second mRNA vaccine dose, the GM NT50 increased to 668 (sd±2.3, median-19.5 fold increase) in these participants. In the saRNA participants with previous infection, baseline GM NT50 was 107 (sd±3.6), increasing to 471 (sd±7.5, median 4.4-fold) two weeks following the second saRNA dose, and to 3,758 (sd±1.9) following the second mRNA vaccine dose (median-6.5 fold). In the non-saRNA group without previous COVID-19 who received mRNA only, three participants were found to have pre-existing neutralising antibodies against Wuhan-hu-1 S at baseline (NT50 322, 171 and 36), despite being negative for binding antibodies and with no clinical history of COVID-19. In the two weeks following the second mRNA vaccine dose, GM NT50 was 2,036 (sd±1.7) in those COVID-19 naïve (NT50 804, 1,862, 1,506 for the three participants who were positive at baseline). In those with previous infection, baseline GM NT50 was 75 (sd±9.3), increasing to 1,980 (sd±3.2) after vaccination (median 27.5-fold change) (Fig 3B). Binding and neutralising antibody titres were significantly higher in the COVAC1 participants with previous infection (Fig 3A and 3B).

The neutralisation capacity against more recent variants of concern (Delta and Omicron variants BA.1 and BA.4/BA.5) was tested 2 weeks following the second mRNA vaccine doses. Only one participant in the non-saRNA previous infection group lost neutralising capacity against BA.1, and four against BA.4/BA.5 (one in each of the non-saRNA groups and two in the saRNA COVID-19 naïve group) with the other participants maintaining some degree of neutralisation (Fig 3C). Overall, neutralisation against variants was best maintained in the saRNA previous infection group following vaccination with mRNA, however this was only statistically significant for BA.4/BA.5 (Fig 3C and 3D). When looking specifically at those with previous infection (regardless of vaccine group) versus those without, there was a median 6-fold reduction in neutralisation against BA.1 versus wildtype, compared to 11-fold for those COVID-19 naïve (p = 0.03), with no other significant differences. Neutralisation was also better maintained against Delta and Omicron variants in the saRNA group versus non-saRNA group regardless of infection status (median 2.8 fold decrease versus 4.8 for Delta, 6.5 vs 10.8 for BA.1, and 21.6 versus 43.3 for BA.4/BA.5) but this was only significant for BA.4/BA.5 (p = 0.003).

A positive correlation was observed between binding antibody and neutralisation titres, with the least correlation seen with neutralisation against BA.4/BA.5 (Fig 3E). Because of the discrepancy between the interval between infection and vaccination in the saRNA and non-saRNA previous COVID-19 groups, we looked to see if there was any correlation between the strength of the antibody response related to time since infection. There was no correlation seen between final binding and neutralising antibody titres against time from infection to first vaccination dose, but there was a weak positive correlation seen against time since infection to the final blood draw for binding (r = 0.43, p = 0.028) but not neutralising antibody titres (S1 Fig). When excluding those participants who had COVID-19 after the first wave, the GM binding and neutralising titres in this group were not significantly different to any other group. The cross-neutralisation against Delta and Omicron variants remained lower in the non-saRNA group compared to the saRNA group, although numbers are small and this was again only significant for BA.4/BA.5 (39.9 fold decrease versus 21.6, p = 0.02)(S2 Fig).

### Cellular responses

**Fig 4:**
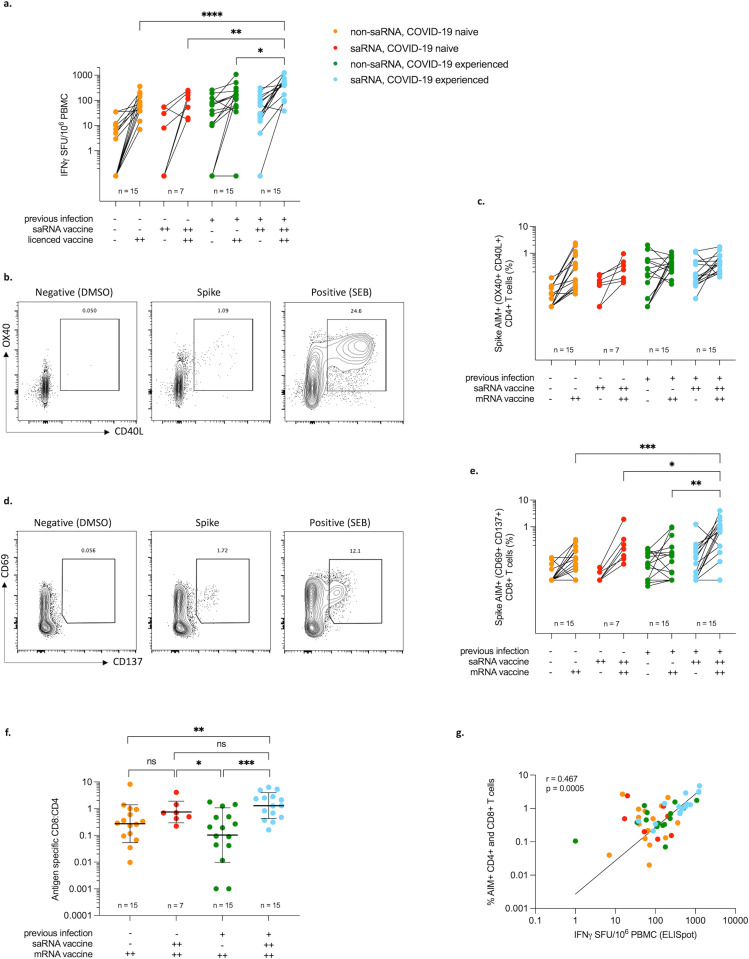
Cellular responses. **4a**. IFN-γ spot forming units (SFU) per million cells (ELISpot) from PBMC stimulated with SARS-CoV-2 spike peptide pools. **4b.** Representative flow cytometry plots of SARS-CoV-2 specific CD4+ cells (CD40L+OX40+) in a vaccinated subject. **4c**. Percentage of SARS-CoV-2 spike specific AIM+ non-naïve CD4+ cells (CD40L+OX40+) at baseline and two weeks following the second licensed vaccine dose in non-saRNA participants (orange and green) and two weeks following the second saRNA and mRNA vaccine doses in saRNA (COVAC1) participants (red and blue) **4d**. Representative flow cytometry plots of SARS-CoV-2 specific CD8+ cells (CD69+CD137+) in a vaccinated subject. **4e**. Percentage of SARS-CoV-2 spike specific AIM+ non-naïve CD8+ cells (CD69+CD137+) at baseline and two weeks following the second mRNA vaccine dose in non-saRNA participants (orange and green) and two weeks following the second saRNA and mRNA vaccine doses in saRNA participants (red and blue). **4f**. Antigen specific CD8+:CD4+ ratio (geometric mean shown). **4g**. Correlation between IFN-γ ELISpot and % antigen specific CD4+ and CD8+ T cells. Graphs are all on a logarithmic scale. Differences between groups determined using Kruskall-Wallis followed by Mann-Whitney for individual comparisons (displayed); Correlation by Spearman’s rank correlation coefficient; -, no exposure; +, single exposure; ++, two exposures; Significant values displayed: *** p<0.005 ** <0.01 *<0.05.

Samples from 14–15 participants from each sub-group were analysed for cellular responses using both IFN-γ ELISpot and an AIM assay. Again participants receiving ChAdOx nCov-19 were excluded from analysis. For those participants in the saRNA group without previous infection (n = 7), the GM spot forming units (SFU) per 10^6^ PBMC was 3 (sd±22.1) two weeks following the second saRNA vaccine dose, increasing to 77 SFU/10^6^ PBMC (sd±3.0) two weeks following the second authorised vaccine dose. In the saRNA participants with previous infection (n = 15), the GM SFU/10^6^ PBMC was 33 (sd±7.1) two weeks following the second saRNA vaccine dose, and 379 SFU/10^6^ PBMC (sd±2.8) following the second mRNA vaccine dose. In the non-saRNA group, the GM SFU/10^6^ PBMC two weeks following the second mRNA dose was 63 (sd±2.7) and 94 (sd±8.1) in those without (n = 15) and with (n = 15) previous infection, respectively (Fig 4A). The number of SFU were higher in the saRNA group with previous infection than in any other group with statistical significance (Fig 4A). The percentage of antigen specific CD4+ T cells following the second authorised vaccine was similar in all groups (Fig 4C), with no significant differences between groups. Six of the non-saRNA previous infection group showed a drop from baseline to post-vaccination (Fig 4C), which could be indicative of the proximity of natural infection to the baseline time-point as this was not seen in any of the participants who were infected in the first wave (S2E Fig). The greatest CD8+ T cell responses were seen in saRNA participants with previous infection 2 weeks following the second authorised vaccine dose (Fig 4E) at GM 0.47% (sd±4.9). This compares to 0.17% (sd±3.6) for the saRNA participants without previous infection and <0.10% for both the non-saRNA groups with and without previous infection. The saRNA groups had the highest proportion of spike specific CD8+ cells relative to CD4+ cells (CD8+:CD4+ ratio GM 0.75 (sd±2.6) and 1.3 (sd±3.1) in COVID-19 naïve and previously infected respectively). This was significant for the saRNA previous COVID-19 group when compared to either non-saRNA groups (CD8+:CD4+ ratio GM 0.3 (sd±5.1) and 0.1 (sd±11.8) in COVID-19 naïve and previously infected respectively) (Fig 4F).

There was a positive correlation seen between IFN-γ release and antigen specific CD4+ and CD8+ T cells (r = 0.47, p = 0.0005) (Fig 4G). Cellular responses as detected by IFN-γ ELISpot and AIM were positively correlated with antibody titres. There was a significant correlation between binding antibody and IFN-γ release (r = 0.56, p = <0.0001) and antigen specific CD4+ T cell responses (r = 0.45, p = 0.0004), and binding antibody and CD8+ responses (r = 0.42, p = 0.002) (S3 Fig). A summary table of the testing results can be seen in Table 2.

**Table 2:** Summary table of experimental results (Geometric means).

	Non-saRNA COVID-19 naive	saRNA COVID-19 naive	Non-saRNA COVID-19 experienced	saRNA COVID-19 experienced
Anti-S IgG (ng/mL)	52,010	40,108	47,917	138,588*
Pseudoneutralisation (NT50)				
Wuhan-hu-1	2,036	595	2,180	3,758*
Delta	584	233	504	1,377*
BA.1	185	68	286	635*
BA.4/5	38	11	37	245*
IFN-γ SFU/10^6^ PBMC	63	77	94	379*
Spike AIM+ CD4+ T cells (%)	0.24	0.23	0.35	0.43
Spike AIM+ CD8+ T cells (%)	0.06	0.17	0.06	0.47*

*statistically significant difference when compared to non-saRNA COVID-19 naïve

## Discussion

This study looks at a unique cohort of individuals, including those that have received a novel COVID-19 saRNA vaccine (COVAC1) followed by a full course of a UK authorised COVID-19 vaccine. Some of these participants have also recovered from COVID-19, thereby having up to five known antigen exposures. The participants that produced the greatest measurable overall antibody and cellular responses were the saRNA group with COVID-19 prior to receipt of any vaccine. These individuals had the greatest quantity of antibody, breadth of neutralisation, and T cell responses as measured by IFN-ƴ ELISpot and AIM assays. It may be expected, and in line with previous publications [18–20], that these convalescent individuals would mount a greater response than those previously naïve to the virus, but we also observed a greater response in these individuals when compared to non-saRNA participants with previous COVID-19 who received two doses of mRNA vaccine only. This could in part be due to the receipt of heterologous vaccines; although looking at different vaccine combinations than used in this study, other studies have observed a better outcome when heterologous vaccines are used for prime-boost [21,22], or as a boost after completion of the original course [23,24]. The COVID-19 experienced saRNA participants also received a total of four vaccine doses, which could be an important factor contributing to the observed difference in the immune response compared to the COVID-19 experienced group that received two doses of mRNA only. It has been demonstrated that there is better breadth of neutralisation, including against Omicron, for individuals receiving three doses of RNA vaccination [25–27]. Data from Israel, where a second booster dose has been introduced, suggests that the boost seen in binding and neutralising antibody titres following the fourth dose was similar to that observed following the third [28] where there was a four month interval between booster doses. However, data from the UK COV-Boost study showed an increase in humoral and cellular responses as measured by ELISA and IFN-γ ELISpots 14 days following the fourth dose compared to 28 days following the third dose of mRNA vaccine in participants who received either ChAdOx nCov-19 or BNT162b2 for their original course [29]. Here, there was a longer interval between third and fourth vaccine doses at a median of 208.5 days [29]. The greater responses we have observed in this study for those who received both saRNA and mRNA following natural infection is likely to be due to a combination of the above factors, including the type of vaccines administered and the total amount of antigen exposures.

Surprisingly, in this study we did not see any significant differences in humoral or cellular responses between those with previous COVID-19 and those without in the non-saRNA group who received mRNA only. This differs from what has been reported elsewhere [18–20,27], and may be a reflection of the relatively small sample size or the interval from infection to vaccination.

Data from the AIM assay showed that participants in the non-saRNA group receiving mRNA vaccination only (both those convalescent and naïve for COVID-19) had greater CD4+ T cell responses than CD8+ T cell responses. Other studies have found an almost universal induction of antigen specific CD4+ T cells following vaccination, with a less consistent induction of CD8+ T cells [30,31], and no significant increase in antigen specific CD8+ T cells following vaccination in convalescent individuals [30]. In contrast, we found that the saRNA participants who received both saRNA and an additional vaccine course had greater CD8+ T cell responses than the non-saRNA group, although this was not significant for the COVID-19 naïve participants. Since the CD4+ T cell response was similar in all four groups, the enhanced CD8+ T cell response seen in the saRNA participants has resulted in an increased ratio of antigen specific CD8+:CD4+ T cells, with the participants with previous infection having a ratio of >1.

Animal models have demonstrated that CD8+ T cells offer protection against SARS-CoV-2 [32,33] even in the absence of detectable antibody [34]. Likewise, in a study of people with haematological malignancy and COVID-19, those with higher CD8+ T cells had a greater survival despite a blunted humoral immune response [35]. Natural infection induces a CD8+ T cell response which is important for viral clearance, and vaccination with RNA somewhat mimics this by expressing viral antigens within the cell which can then use MHC class I machinery. Although findings are variable, overall, studies in humans have found a greater and more consistent CD4+ than CD8+ T cell response following mRNA vaccination [17,36,37]. It may be that the different kinetics with saRNA versus mRNA vaccines generate a greater CD8+ T cell response, perhaps due to ongoing antigen expression. Murine models looking at saRNA vaccination have demonstrated a greater CD8+ T cell response when compared to other vaccine platforms despite a similar CD4+ T cell response [38,39], with the greatest responses seen when heterologous vaccines are used [40,41]. Despite the small numbers in our study, we have observed a similar pattern of increased CD8+ T cell responses in humans following saRNA vaccination which has perhaps been enhanced by hybrid immunity.

It is important to note that the study population and comparison groups are quite heterogenous, and that the sample size is small. One such difference that could be important when considering the interpretation of the results is the timing of infection for those participants with previous COVID-19. All participants with previous COVID-19 in the saRNA group were infected during the first wave (Wuhan-hu-1), whereas two thirds of those in the non-saRNA group were infected during the second wave when the Alpha variant (B.1.1.7.) was dominant, and these participants have a much shorter interval between infection and vaccination. This reflects the timing of recruitment as COVAC1 participants were recruited earlier (August 2020) than the non-saRNA group who were only recruited after COVID-19 vaccines became available (Dec 2020-January 2021). This could be contributing to the higher response seen in the saRNA previous infection group compared to the non-saRNA group. A weak positive correlation was observed between the binding antibody titres and days since infection to the final blood draw, however, no significant differences were seen in binding or neutralising antibody titres between those infected in wave one versus wave two within this non-saRNA group. It has also been suggested that infection with Alpha may result in a reduction in neutralisation of variants compared to infection with Wuhan-hu-1 [42–44]. This could also be in part due to a shorter interval between infection and subsequent repeat antigen exposure. However, it has also been demonstrated that for those infected with Alpha, neutralisation against wildtype Wuhan-hu-1 is retained, and there is still potent cross-neutralising activity [44]. In our study, when the participants in the non-saRNA group with previous COVID-19 in the second wave were excluded, there were no significant differences in the breadth of neutralisation against Delta and Omicron variants between this group and any other group (S2C Fig). There remained a trend towards a higher breadth of neutralisation in the saRNA group, although total numbers are small.

There were also differences seen in the characteristics of the participants who were COVID-19 naïve compared to those with previous infection–this was evident in the saRNA cohort, particularly for those participants who went on to receive ChAdOx nCoV-19 rather than BTN162b2. These participants were more likely to be older and male than the other groups and were also less likely to be healthcare workers, and therefore less likely to have ongoing exposures to COVID-19. This may be significant when interpreting the results in this cohort in comparison to the others as it has been shown that age may be an important factor for the generation of a vaccine response [45–47]. Over 50% of this group received the ChAdOx nCoV-19 vaccine, for which the post vaccine timepoints have been excluded from the analysis. Although this then leaves a very small sample size in this group, removal of these participants from the analysis means that the differences in age, sex and healthcare worker status are not significantly different between groups.

The findings from this study suggest an immunological benefit of increased antigen exposures which included saRNA in this study. This was particularly evident in the convalescent individuals receiving heterologous saRNA and mRNA vaccination. Although the effects of saRNA vaccination alone were limited in comparison to available vaccine platforms, these participants did appear to have immune priming which was then robustly boosted by the receipt of BNT162b2. The findings from this study would support the potential for future iterations of this saRNA vaccine to be used in combination with other vaccine platforms or as a booster in those previously antigen exposed, both in the context of COVID-19 and other infectious pathogens.

## Materials and methods

### Ethics statement

COVAC1 was approved in the UK by the Medicines and Healthcare products Regulatory Agency and the North East–York Research Ethics Committee (reference 20/SC/0145) (ISRCTN17072692, EudraCT 2020-001646-20).

The recruitment of participants and the collection and storage of samples into the Imperial College London Communicable Diseases Research Tissue Bank (NRES ID 20/SC/0226) was approved by the Tissue Bank Steering Committee. Samples were donated following written informed consent.

### Participants

COVAC1 participants in the expanded safety evaluation cohort at ICHT gave written informed consent to be recruited into a sub-study. Blood sampling for serum (6 mL) and PBMC separation (up to 40 mL) were taken at multiple time points out to one year (Fig 1). Following the initiation of the NHS COVID-19 vaccine programme in December 2020, COVAC1 participants received vaccination with a UK authorised COVID-19 vaccine. Sub-study participants were consented to donate additional blood samples for serum and PBMC following each authorised vaccination dose. These samples were collected into a pre-existing tissue bank at Imperial College London (the Communicable Diseases Research Tissue Bank).

Participants receiving only authorised COVID-19 vaccination were recruited into a comparison group (non-saRNA) from the ICHT vaccine hub. Participants provided written informed consent for samples to be collected into the tissue bank as above, and blood samples for serum and PBMC were collected at intervals pre- and post- each vaccine dose (Fig 1).

Participants were asked about history of COVID-19, defined as a previous PCR positive or positive antibody test on an approved platform. For those with previous infection, data were collected on dates of illness, disease severity and symptoms. Previous infection was additionally defined as the presence of SARS-CoV-2 S or N antibodies at baseline as detected by ELISA.

### Sample processing and storage

Sera were spun, separated, and heat inactivated for 30 minutes at 56°C prior to storage at -80°C. Samples were thawed prior to immunological assessment.

Blood for PBMC separation was collected in sodium heparin tubes and processed within 4 hours of collection. Samples were separated using leucosep tubes prepared with Histopaque. The PBMC layer was then poured into a new falcon, washed twice and counted using an automatic cell counter (Vi-Cell). Samples were stored in freezing media (10% DMSO/90% FCS) at a concentration of 1x10^7^ PBMC/mL in 1 mL aliquots at -150°C after being kept overnight in a Stratacooler at -80°C.

### SARS-CoV-2 anti-S and anti-N ELISA

Binding IgG concentrations against SARS-CoV-2 spike (S) and nucleocapsid (N) proteins were quantified using ELISA. 96-well binding plates were coated with recombinant SARS-CoV-2 S or N protein for sample wells, and capture antibody (anti-human kappa and lambda light chain specific mouse antibody) for standard wells. After 1 hour at 37°C, plates were washed and blocked for 1 hour with 2% Bovine serum albumin (BSA) before the addition of sera in triplicate starting at a 1:50 dilution to the antigen coated wells, and a serial dilution of 1:5 of purified human IgG standards in triplicate to the kappa/lambda antibody coated wells, followed by a 1 hour incubation at 37°C. Secondary antibody HRP conjugated goat anti-human IgG was added and the plates incubated for a further hour before developing with TMB substrate. The reaction was stopped after 5 minutes by adding TMB stop solution and the absorbance read at 450 nm on a VersaMax plate reader. For low level antibody detection, a sensitive IgG ELISA was used to test participant sera which included biotin-conjugated anti-human IgG and streptavidin-HRP [16].

The optical density (OD) 450 nm values obtained from the known concentrations of human IgG antibody standard were used to generate a standard curve from which the ODs from the serum samples can be given a concentration in ng/ml of spike-specific IgG. The data were expressed as positive if the blank-subtracted OD was above the pre-determined cut-off of OD 0.2 nm and values were on the linear portion of the standard curve. This OD value of 0.2 was obtained during assay optimisation using standards from National Institute for Biological Standards and Control (NIBSC), convalescent sera from those recovered from COVID-19, and pre-pandemic sera. On every plate, two positive controls (a WHO international standard and NIBSC positive control) were run in triplicate along with a negative control (pre-pandemic sera) the values of which must be consistent. Analysis of the data were performed using SoftMax Pro GxP software.

### Pseudoneutralisation assay

Pseudotyped SARS-CoV-2 lentiviruses were produced in HEK293T/17 cells using a SARS-CoV-2 spike plasmid, HIV-1 gag-pol plasmid and a firefly luciferase reporter. SARS-CoV-2 spike plasmid for Wuhan-hu-1 (wildtype), B.1.617.2 (Delta) and B.1.1.529 (Omicron variants BA.1 and BA.4/BA.5) were obtained from the Wendy Barclay laboratory at Imperial College London. Serum samples were serially diluted and incubated in a 96 well plate with pseudovirus (PSV) for 1 hour. HEK293T-ACE2 cells were then added at 1x10^4^ cells/well and incubated for up to 96 hours. The luciferase expression was then measured in infected HEK293T-ACE2 cells after the addition of Bright-Glo Luciferase working solution to each well using the BMG Labtech Omega Fluorostar luminometer. NT50 neutralisation titres were calculated as the dilution at which relative luminescence was reduced by 50% compared with control. The WHO international standard positive control and a pre-pandemic negative control was run with each experiment and NT50 values needed to remain consistent.

### Peptide pools for stimulation studies

Three peptide pools were made containing a total of 316 peptides, each of 15 amino acids in length, overlapping by 11 residues (PP1 = 100 peptides, PP2 = 100 peptides and PP3 = 116 peptides) covering the SARS-CoV-2 S protein sequence from the COVAC1 vaccine. The peptide pools were reconstituted in sterile water and DMSO with a final concentration of 20 μg/mL with DMSO at 1.9%, 1.9% and 2.2% in each peptide pool, respectively. This gives a final concentration of 0.2% when used in the ELISpot assay and 0.15% for the AIM assay.

### ELISpot assay

An Enzyme-Linked Immunosorbent Spot (ELISpot) assay was used to detect IFN-ƴ secreting T cells from stimulated PBMC in 14–15 participants from each subgroup. These participants were selected based on the availability of stored PBMCs. Participants receiving ChAdOx nCoV-19 were excluded from analysis, leaving the results from seven participants reported in the saRNA COVID-19 naïve group. Cells were stimulated with each of the three spike peptide pools, a negative control (media) and two positive controls (anti-CD3 and CEFX). Cryopreserved cells were thawed, resuspended in media and incubated at 37°C, 5% CO_2_ overnight. Stimulation media was added onto plates pre-coated with anti-human IFN-ƴ capture antibody that were washed with PBS and blocked with R10 (RPMI with 10% fetal calf serum). Cells were counted (Vi-Cell automated cell counter) and added in triplicate at 2 x 10^5^ cells/well, and plates were incubated at 37°C for 16–24 hours. Post incubation, plates were washed, and mouse anti-human IFN-ƴ detection antibody was added prior to a 2 hour incubation at room temperature. The plates were washed again, streptavadin-ALP was added, and the plates were incubated for a further 1 hour. After a further wash, BCIP/NBT was added for 5–15 minutes until spots developed. The reaction was then stopped and the plates left to dry in the dark before being read on the ELISpot reader (AID iSpot Automated Reader). The mean number of spot forming units (SFU) for each condition was calculated and the background from the negative control wells subtracted. The results for the three peptide pools are summated to give the total SFU/million PBMC for each sample analysed.

### AIM assay

Cryopreserved PBMC were thawed and resuspended into AIM-V media. Cells were counted and plated at a concentration of 1–2 x 10^6^ PBMC/well. They were then left to rest for 3 hours at 37°C, 5% CO_2_. Following incubation, CD40 blocking antibody and antibodies that may be downregulated were added (CXCR5 and CCR7). These were then incubated for a further 15 minutes before incubation with either the combined SARS-CoV-2 spike peptide pools, a positive control (staphylococcal enterotoxin B, SEB) or negative control (AIM-V with 0.15% DMSO) stimulation media. After a 24-hour stimulation, the cells were stained with the viability dye for 20 minutes and the remaining antibodies for 30 minutes before fixation with 1% paraformaldehyde. The full panel can be seen in S1 Table. The samples were then analysed using the Aurora spectral flow cytometer. Raw samples from the Aurora were unmixed on Spectroflo using a combination of controls recorded from cells and beads. Percentage of non-naïve AIM+ CD4+ and CD8+ cells were calculated (S4 Fig for gating strategy). AIM+ CD4+ cells were defined as CD40L+ OX40+ and AIM+ CD8+ cells as CD69+ CD137+.

### Data collection and analysis

Data were analysed using GraphPad Prism version 9.3.1 and FlowJo version 10.8.1. Non-parametric statistical tests were used throughout including the Kruskal-Wallis and Mann Whitney U tests to compare groups, Wilcoxon matched pairs signed rank test for within group paired comparisons, and the Spearman correlation coefficient to assess the strength of association between variables.

## Supporting information

S1 Fig S1a. Correlation between time from infection to first vaccination dose and final measured binding antibody titres (two weeks following second UK authorised vaccine dose). S1b. Correlation between time from infection to first vaccination dose and final measured neutralising antibody titres. S1c. Correlation between time from infection to last blood draw (two weeks following second authorised vaccine dose) and binding antibody titres. S1d. Correlation between time from infection to last blood draw and neutralising antibody titres. Graphs on logarithmic scale. Correlation by Spearman’s rank correlation coefficient.
(TIF)

S2 Fig Humoral and cellular immune responses excluding participants with previous COVID-19 after wave 1. **S2a.** Antibodies against Wuhan-hu-1 spike protein as measured by ELISA at baseline and two weeks following dose 1 and 2 of saRNA and mRNA vaccines in saRNA (COVAC1) participants (red and blue) and at baseline and two weeks following dose 1 and 2 of mRNA vaccines in non-saRNA participants (orange and green). **S2b.** Neutralising antibodies against SARS-CoV-2 Wuhan-hu-1 measured using pseudovirus at baseline and two weeks following the 2^nd^ dose of saRNA and mRNA vaccines in saRNA participants (red and blue) and two weeks following the 2^nd^ dose of mRNA vaccine in non-saRNA participants (orange and green). **S2c.** Neutralising antibodies against SARS-CoV-2 Wuhan-hu-1, Delta and Omicron variants BA.1 and BA.4/5 using pseudovirus two weeks following the second mRNA vaccine dose, and the fold decrease of neutralisation of variants compared to Wuhan-hu-1. **S2d.** IFN-ƴ spot forming units (SFU) per million cells (ELISpot) from PBMC stimulated with SARS-CoV-2 spike peptide pools. **S2e.** Percentage of SARS-CoV-2 spike specific AIM+ non-naïve CD4+ cells (CD40L+OX40+) at baseline and two weeks following the second mRNA vaccine dose in non-saRNA participants (orange and green) and two weeks following the second saRNA and mRNA vaccine doses in saRNA (COVAC1) participants (red and blue). **S2f.** Percentage of SARS-CoV-2 spike specific AIM+ non-naïve CD8+ cells (CD69+CD137+) at baseline and two weeks following the second mRNA vaccine dose in non-saRNA participants (orange and green) and two weeks following the second saRNA and mRNA vaccine doses in saRNA participants (red and blue). **S2g.** Antigen specific CD8+:CD4+ ratio. Graphs are all on a logarithmic scale. Geometric mean and standard deviations are shown Differences between groups determined using Kruskall-Wallis followed by Mann-Whitney for individual comparisons. Median fold decrease within groups by Wilcoxon matched pairs signed rank test. LOD, level of detection; -, no exposure; +, single exposure; ++, two exposures; significance values: **** p<0.0001; *** p<0.001; ** p<0.01; * p<0.05; ns, non-significant; WT, wildtype; Δ, Delta.
(TIF)

S3 Fig S3a.Correlation between IFN-g spot forming units (SFU)/106 PBMC stimulated with SARS-CoV-2 spike (S) peptide pools and binding antibodies against SARS-CoV-2 S protein. S3b. Correlation between percentage of antigen specific CD4+ T cells (CD40L+ OX40+) and binding antibodies against SARS-CoV-2 S protein. S3c. Correlation between percentage of antigen specific CD8+ T cells (CD69+ CD137+) and binding antibodies against SARS-CoV-2 S protein. Graphs on logarithmic scale. Correlation by Spearman’s rank correlation coefficient.
(TIF)

S4 Fig Gating strategy for activation induced marker (AIM) assay to detect non-naïve antigen specific CD4+ and CD8+ T cells from peripheral blood mononuclear cells (PBMCs) stimulated with SARS-CoV-2 spike protein peptide pools. (TIF)

S1 Table Activation induced marker panel. (TIF)
