# Supplementary figures and images for: Enhanced immune responses following heterologous vaccination with self-amplifying RNA and mRNA COVID-19 vaccines
Source: PLoS Pathog. 2022 Oct 4;18(10):e1010885. doi: 10.1371/journal.ppat.1010885 (PMC9565686; doi:10.1371/journal.ppat.1010885)

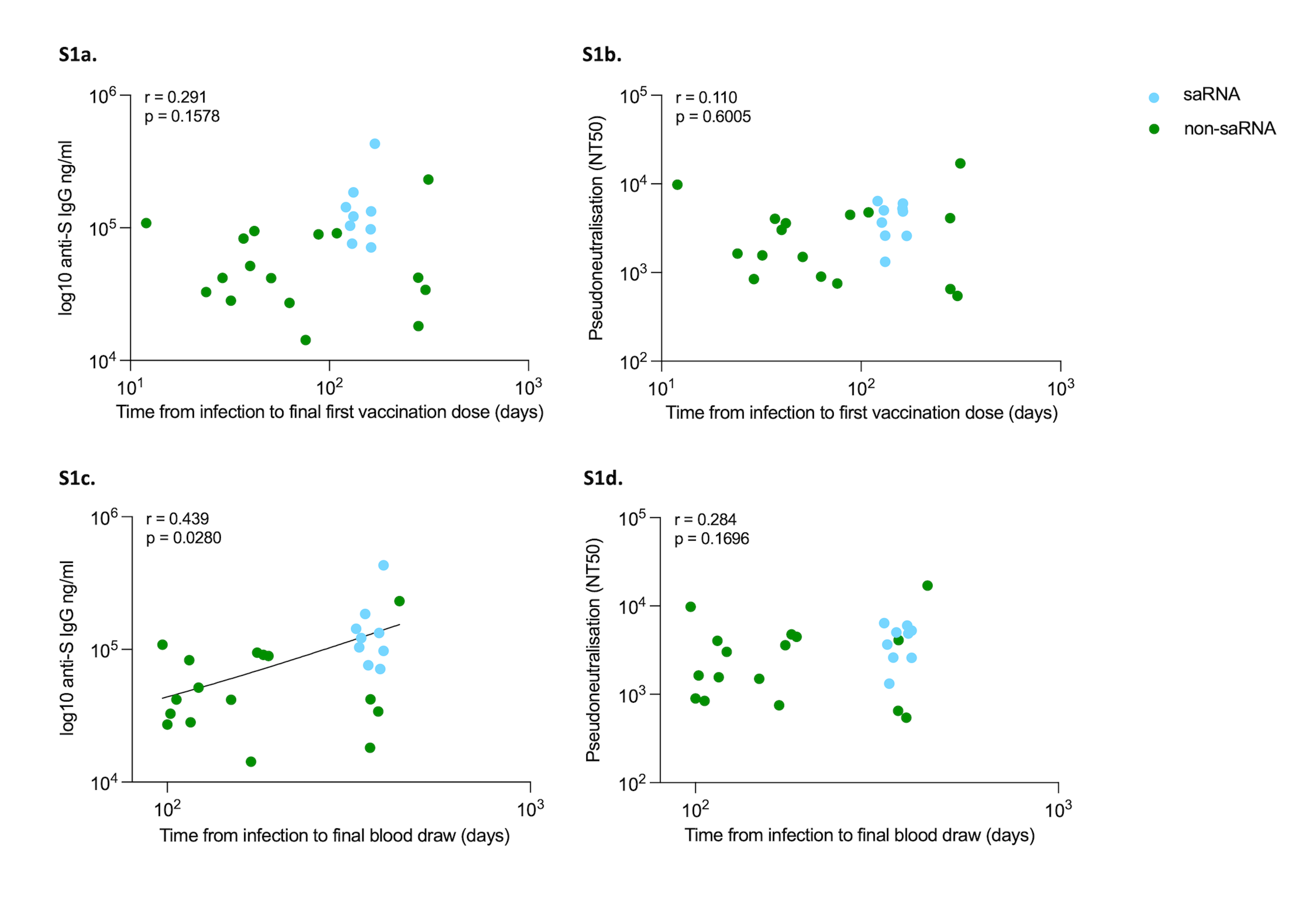

Supplement: S1 Fig — S1a. Correlation between time from infection to first vaccination dose and final measured binding antibody titres (two weeks following second UK authorised vaccine dose). S1b. Correlation between time from infection to first vaccination dose and final measured neutralising antibody titres. S1c. Correlation between time from infection to last blood draw (two weeks following second authorised vaccine dose) and binding antibody titres. S1d. Correlation between time from infection to last blood draw and neutralising antibody titres. Graphs on logarithmic scale. Correlation by Spearman’s rank correlation coefficient. (TIF) [file ppat.1010885.s001.tif]

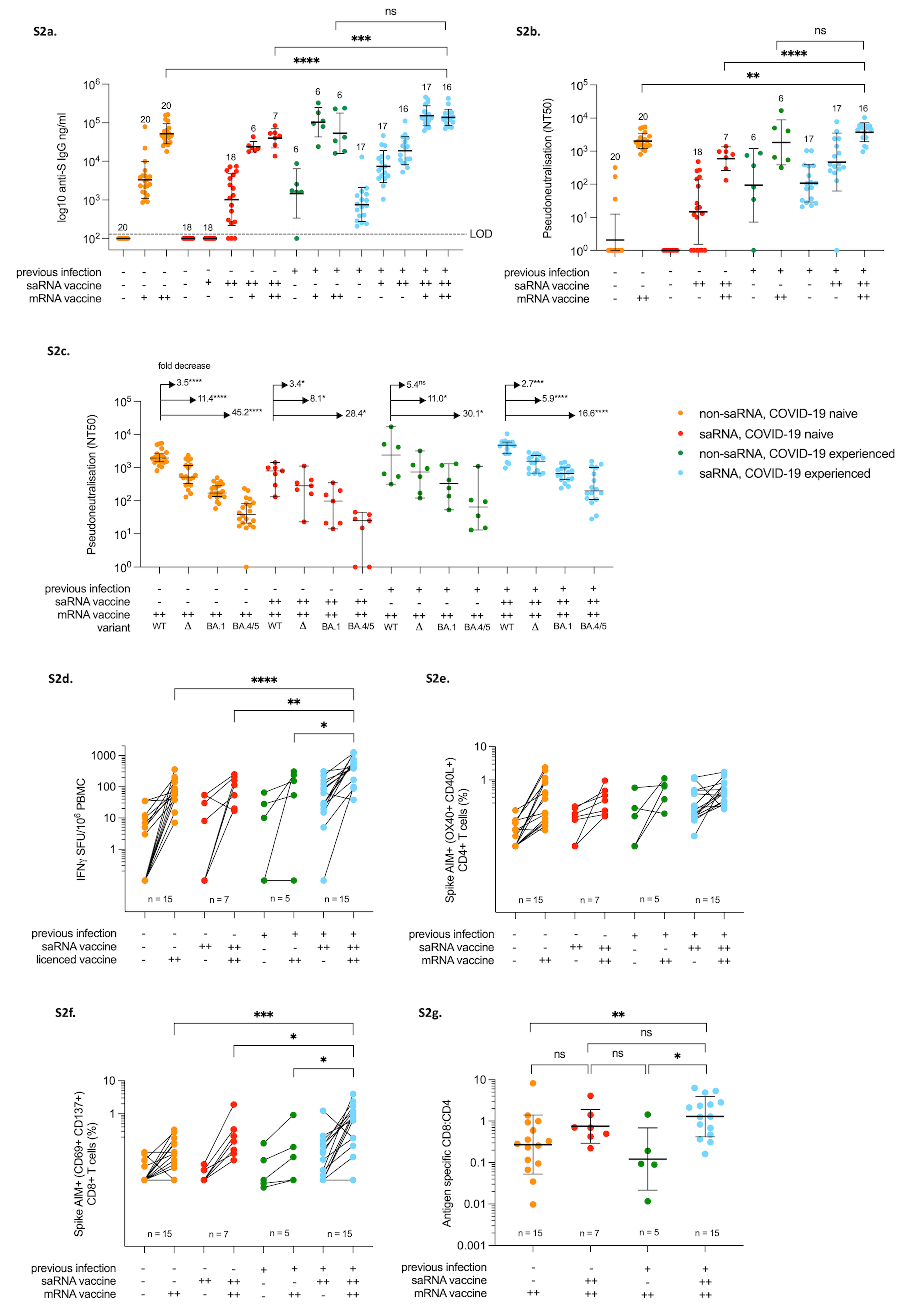

Supplement: S2 Fig — S2a. Antibodies against Wuhan-hu-1 spike protein as measured by ELISA at baseline and two weeks following dose 1 and 2 of saRNA and mRNA vaccines in saRNA (COVAC1) participants (red and blue) and at baseline and two weeks following dose 1 and 2 of mRNA vaccines in non-saRNA participants (orange and green). S2b. Neutralising antibodies against SARS-CoV-2 Wuhan-hu-1 measured using pseudovirus at baseline and two weeks following the 2nd dose of saRNA and mRNA vaccines in saRNA participants (red and blue) and two weeks following the 2nd dose of mRNA vaccine in non-saRNA participants (orange and green). S2c. Neutralising antibodies against SARS-CoV-2 Wuhan-hu-1, Delta and Omicron variants BA.1 and BA.4/5 using pseudovirus two weeks following the second mRNA vaccine dose, and the fold decrease of neutralisation of variants compared to Wuhan-hu-1. S2d. IFN-ƴ spot forming units (SFU) per million cells (ELISpot) from PBMC stimulated with SARS-CoV-2 spike peptide pools. S2e. Percentage of SARS-CoV-2 spike specific AIM+ non-naïve CD4+ cells (CD40L+OX40+) at baseline and two weeks following the second mRNA vaccine dose in non-saRNA participants (orange and green) and two weeks following the second saRNA and mRNA vaccine doses in saRNA (COVAC1) participants (red and blue). S2f. Percentage of SARS-CoV-2 spike specific AIM+ non-naïve CD8+ cells (CD69+CD137+) at baseline and two weeks following the second mRNA vaccine dose in non-saRNA participants (orange and green) and two weeks following the second saRNA and mRNA vaccine doses in saRNA participants (red and blue). S2g. Antigen specific CD8+:CD4+ ratio. Graphs are all on a logarithmic scale. Geometric mean and standard deviations are shown Differences between groups determined using Kruskall-Wallis followed by Mann-Whitney for individual comparisons. Median fold decrease within groups by Wilcoxon matched pairs signed rank test. LOD, level of detection; -, no exposure; +, single exposure; ++, two exposures; significance values: ** [file ppat.1010885.s002.tif]

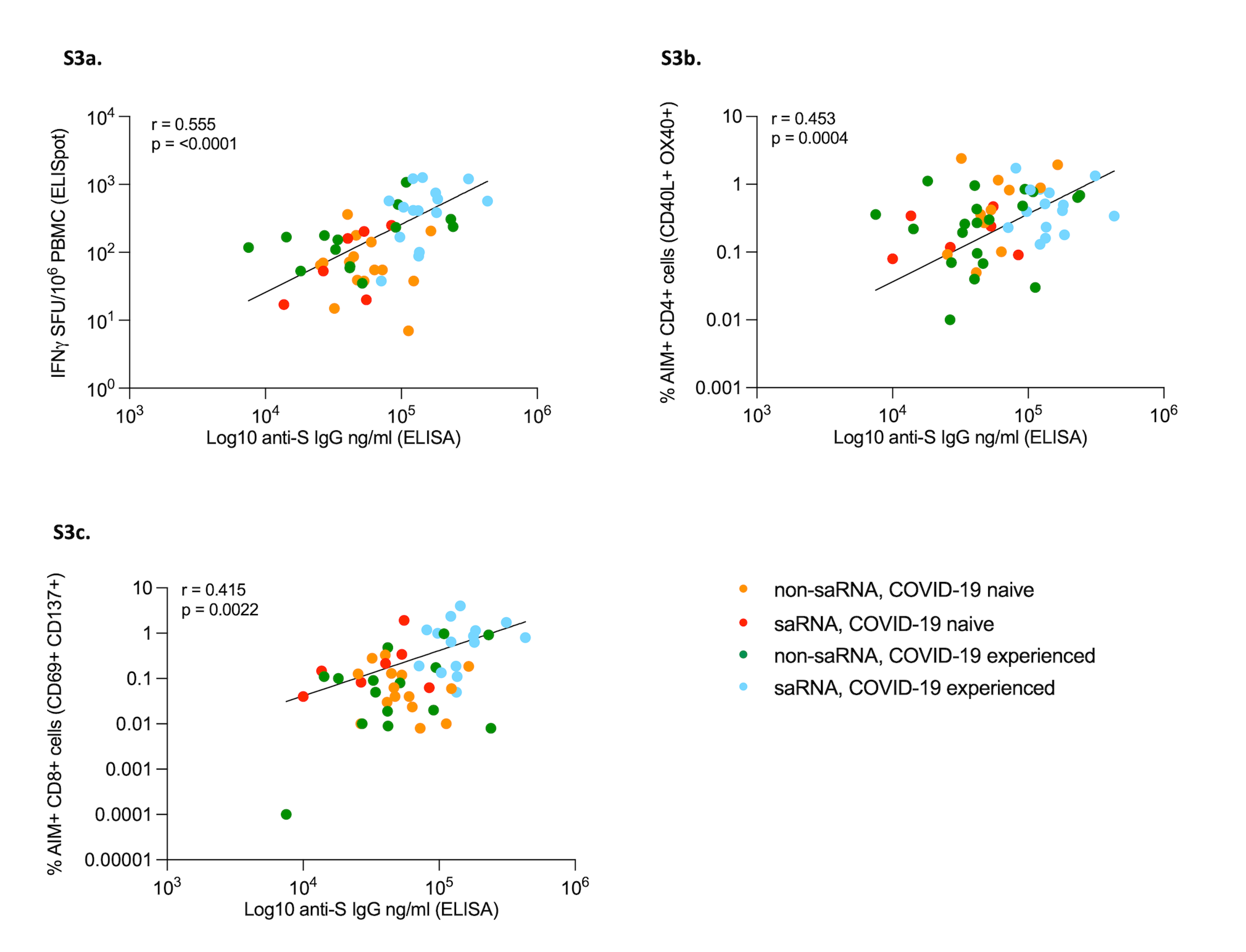

Supplement: S3 Fig — S3a.Correlation between IFN-g spot forming units (SFU)/106 PBMC stimulated with SARS-CoV-2 spike (S) peptide pools and binding antibodies against SARS-CoV-2 S protein. S3b. Correlation between percentage of antigen specific CD4+ T cells (CD40L+ OX40+) and binding antibodies against SARS-CoV-2 S protein. S3c. Correlation between percentage of antigen specific CD8+ T cells (CD69+ CD137+) and binding antibodies against SARS-CoV-2 S protein. Graphs on logarithmic scale. Correlation by Spearman’s rank correlation coefficient. (TIF) [file ppat.1010885.s003.tif]

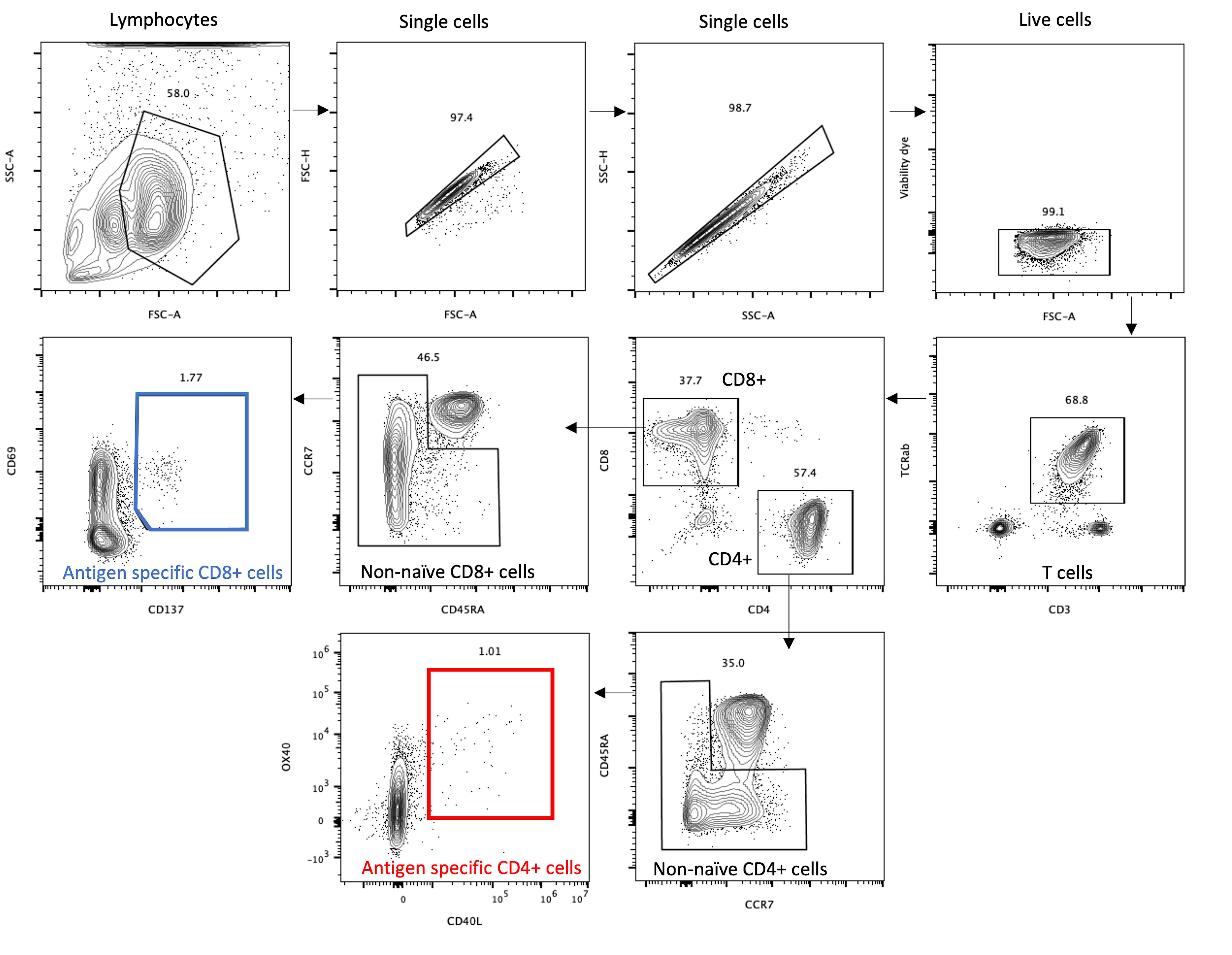

Supplement: S4 Fig — (TIF) [file ppat.1010885.s004.tif]

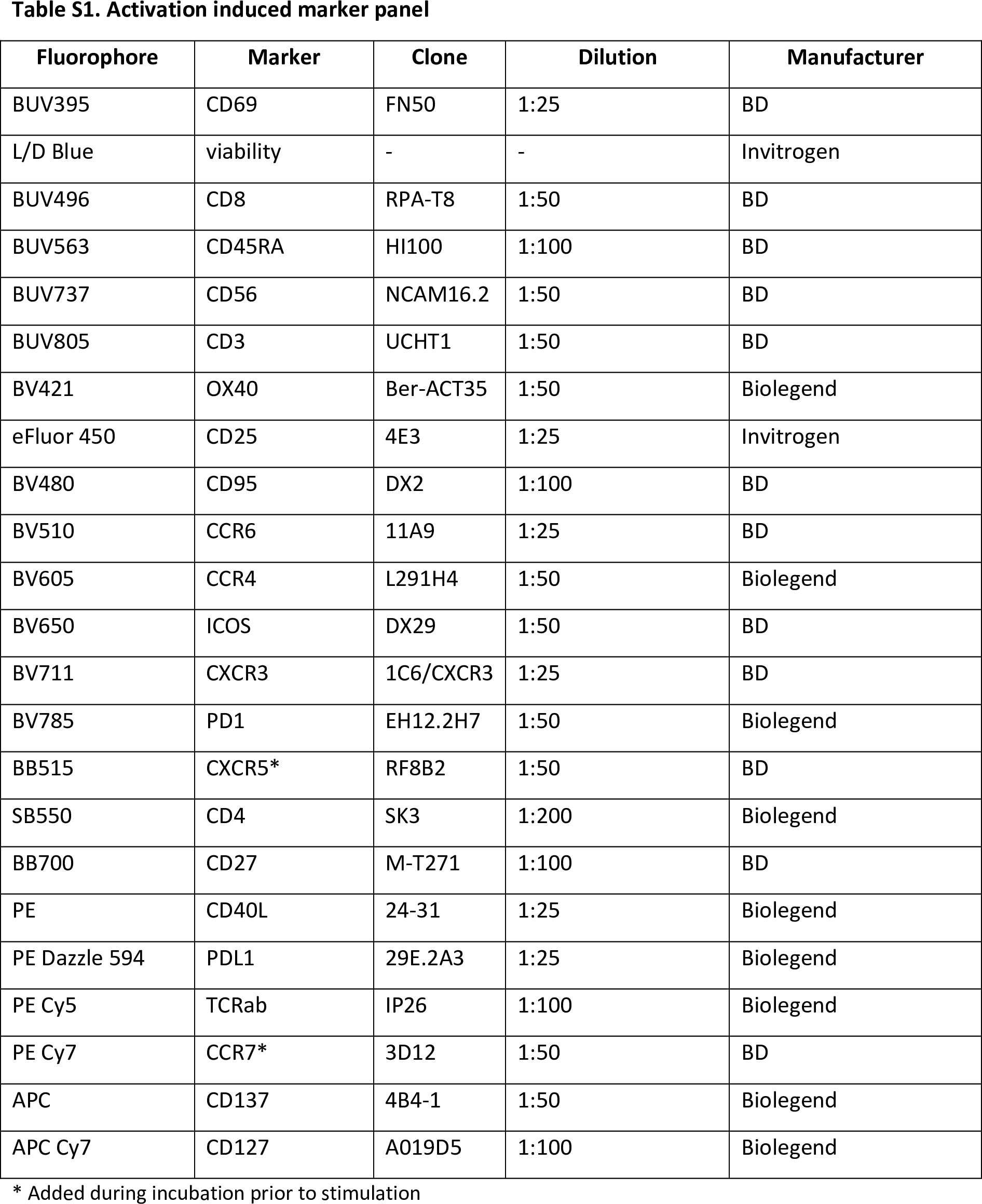

Supplement: S1 Table — (TIF) [file ppat.1010885.s005.tif]
